# Redox control of glutamine utilization in cancer

**DOI:** 10.1038/cddis.2014.513

**Published:** 2014-12-04

**Authors:** L Alberghina, D Gaglio

**Affiliations:** 1 SYSBIO Center for Systems Biology, Department of Biotechnology and Biosciences, University of Milano-Bicocca, Milan and Rome, Italy; 2 Department of Biotechnology and Biosciences, University of Milano-Bicocca, Piazza della Scienza 2, Milan, Italy; 3 Institute of Molecular Bioimaging and Physiology (IBFM), National Research Council (CNR), Via F.lli Cervi 93, Segrate, Milan, Italy

## Abstract

Glutamine utilization promotes enhanced growth of cancer cells. We propose a new concept map of cancer metabolism in which mitochondrial NADH and NADPH, in the presence of a dysfunctional electron transfer chain, promote reductive carboxylation from glutamine. We also discuss why nicotinamide nucleotide transhydrogenase (NNT) is required *in vivo* for glutamine utilization by reductive carboxylation. Moreover, NADPH, generated by both the pentose phosphate pathway and the cancer-specific serine glycolytic diversion, appears to sustain glutamine utilization for amino-acid synthesis, lipid synthesis, and for ROS quenching. The fact that the supply of NAD^+^ precursors reduces tumor aggressiveness suggests experimental approaches to clarify the role of the NADH-driven redox network in cancer.

## Facts

- Sustained growth and survival of most cancer cells rely upon a metabolic rewiring, characterized by an enhanced glycolytic flux and a stimulated utilization of glutamine by reductive carboxylation.
- Mitochondrial dysfunction (for instance Complex I reduced activity) facilitates insurgence of cancer metabolic rewiring.
- NAD^+^/NADH and NADP^+^/NADPH are involved in several reactions of the cancer metabolic rewiring pathways.
- Manipulation of NAD(H) metabolism in cancer cells affects their tumor forming ability in xenografts.

## Open Questions

- Which is, in various cancer cell types characterized by differential aggressiveness, the quantitative flux of NADPH (coming either from the pentose phosphate pathway or from the serine synthesis, one-carbon-folate- metabolism and glycine cleavage pathway) that ends up in NEAA, in GSH, and in lipids?
- Which is the mitochondrial/cytosolic compartmentalization of NADH and of NADPH metabolism in cancer metabolic rewiring?
- Does the cytoplasmic malate dehydrogenase/malic enzyme pathway convert NADH to NADPH in cancer cells?

Two metabolic adaptations have been recognized as hallmarks of cancer cells: (1) an increased utilization of glucose to produce lactate through the glycolytic pathway^1^ and (2) an enhanced consumption of glutamine by reductive carboxylation to sustain anabolic processes.^2^ Although the metabolic profile of cancer cells may be affected by the tissue that is transformed and by the responsible oncogene,^3^ a large number of different cancer types present the indicated metabolic rewiring. It follows that a glucose analog, 2-fluoro-deoxy-glucose (FDG), is currently employed in positron emission tomography (PET) for diagnostic purposes.^4^

Several oncogenic signaling pathways, often dependent upon the activation of oncogenes such as ras and myc, found mutated in several human cancers,^5, 6, 7^ express their transforming activity by inducing mitochondrial dysfunction (which often causes a reduction of Complex I activity) and by promoting glycolysis and glutamine utilization.^6, 8, 9, 10, 11, 12, 13, 14, 15, 16, 17^

Inhibition of the OXPHOS mitochondrial pathway, and more specifically of Complex I, is able to promote glutamine utilization.^18^ Both pathways of cancer metabolic remodeling are required to support cancer cell proliferation. In fact both inhibition of mitochondrial glutaminase (GLS2), which catalyzes the hydrolysis of glutamine to glutamate, and inhibition of lactate dehydrogenase A, the enzyme that converts pyruvate into lactate, are able to suppress tumor cell proliferation.^19, 20^ No information is presently available on links between glycolysis and glutamine utilization by reductive carboxylation, although it has been reported that, with few exceptions,^21, 22^ cancer cell growth cannot proceed in the presence of only glutamine, with no glucose present.^23^ Taken together, these findings have suggested that metabolic differences of various cancer cells types may open new ways of target identification towards more effective anti-cancer drugs.^24, 25^

To reach this aim, it is of course necessary to clarify in detail the cancer-specific metabolic pathways. In this paper, we present a reconstruction of the glutamine and glucose utilization pathways in cancer cells which, in a novel manner, clarifies their regulatory connections with red/ox processes centered on NAD^+^/NADH and NADP^+^/NADPH. No coverage is given here on the manifold and complex relationship between pathways of NAD(P)^+^ utilization and on their impact on glutamine and glucose metabolism. The NAD(P)^+^-utilizing systems include (i) mono ADP-ribosylation reactions, (ii) Poly ADPR polymerases (PARPs), (iii) ADP ribosyl cyclases, and (iv) sirtuins.

## Pathways of Glutamine Utilization in Cancer Cells

**Figure 1:**
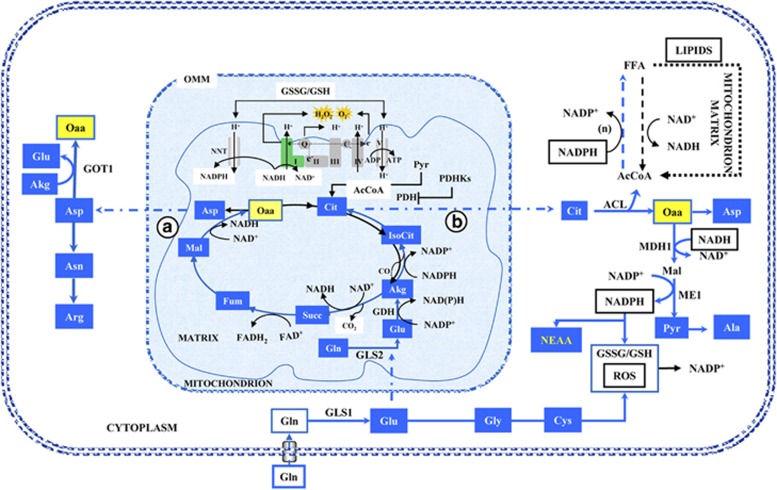
Schematic representation of glutamine metabolic rewiring. Glutamine imported *via* Slc1a5 enters in a complex metabolic pathway, described in the text, so that both its carbon and nitrogen are utilized to promote growth and survival of cancer cells. AcCoA, acetyl-CoA; ACL, ATP citrate lyase; Akg, *α*-ketoglutarate; Ala, alanine; Arg, arginine; Asn, asparagine; Asp, aspartate; Cit, citrate; Cys, cysteine; FFA, fatty acids; Fum, Fumarate; GDH, glutamate dehydrogenase; Gln, glutamine; Glu, glutamate; Gly, glycine; GOT, glutamic-oxaloacetic transaminase; GSH, glutathione reduced; GSSG, glutathione oxidized; Isocit, isocitrate; Mal, malate; MDH 1 and 2, malate dehydrogenase; ME, malic enzyme; NEAA, non-essential amino acids; NNT, nicotinamide nucleotide transhydrogenase; Oaa, oxalacetate; PDH, pyruvate dehydrogenase; PDHKs, pyruvate dehydrogenase kinases; Pyr, pyruvate; ROS, reactive species oxygen; Succ, succinate

**Figure 2:**
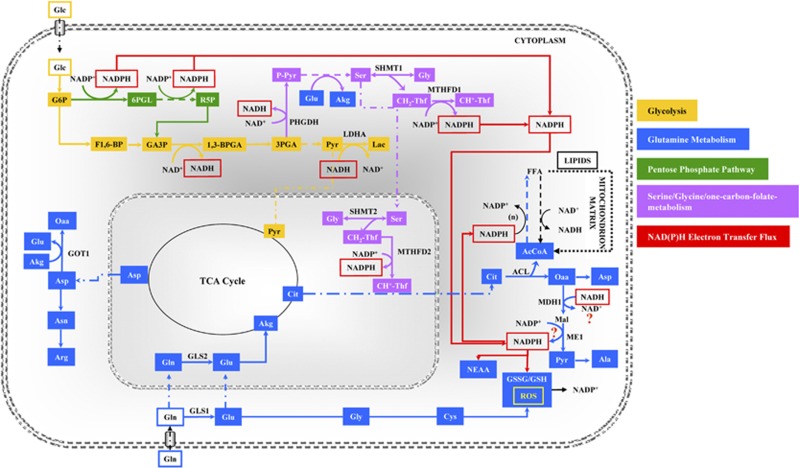
Concept map of the cancer metabolic rewiring. Large glycolytic flux and complex I dysfunction are required to sustain glutamine reductive carboxylation. Yellow arrows identify glycolysis. Blue arrows identify glutamine metabolism, green arrows identify pentose phosphate pathway (PPP), bright Lilac identifies serine/glycine/one-carbon-folate-metabolism, and red arrows identify an NAD(P)H electron transfer flux (ETF). NAD(P)H ETF originates from serine diversion pathway, sustains lipid synthesis, ROS quenching by GSH and reductive steps of non-essential amino-acid (NEAA) synthesis. Abbreviations: 1,3BPGA, bisphosphoglycerate; 3PGA, 3phosphoglycerate; 6PGL, 6-phosphogluconolactone; AcCoA, acetyl-CoA; ACL, ATP citrate lyase; Akg, *α*-ketoglutarate; Ala, alanine; Arg, arginine; Asn, asparagine; Asp, aspartate; CH^+^-Thf, 5-methylenetetrahydrofolate; CH_2_-Thf, 5,10-methylenetetrahydrofolate; Cit, citrate; Cys, cysteine; F1,6BP, fructose 1,6 biphosphate; FFA, fatty acids; G6P, glucose 6-phosphate; GA3P, glyceraldehyde 3-phosphate; Glc, glucose; Gln, glutamine; Glu, glutamate; Gly, glycine; GOT, glutamic-oxaloacetic transaminase; GSH, glutathione reduced; GSSG, glutathione oxidized; Lac, lactate; LDHA, lactate dehydrogenase; MDH 1, malate dehydrogenase; ME, malic enzyme; MTHFD1 and 2, methylenetetrahydrofolate dehydrogenase (NADP^+^ dependent); NEAA, non-essential amino acids; Oaa, oxalacetate; PPP, pentose phosphate pathway; P-Pyr, 3-phosphohydroxypyruvate; Pyr, pyruvate; R5P, ribose 5-phosphate; ROS, reactive species oxygen; Ser, serine; SHMT1 and 2, serine hydroxymethyltransferase

The network of biochemical reactions of mammalian cell metabolism is well known and genome-wide reconstructions of human metabolism, specific for many cell types, are available.^26^ Recently, the role of signaling in controlling metabolism by affecting enzyme activities either at the transcriptional or at the translational and the post-translational levels has been elucidated in a number of physio/pathological conditions.^27^ Metabolic maps are available that annotate the gene encoding for any given enzyme, the kinetic parameters for each substrate, the required co-factors, the allosteric regulators, but the simple measurement of enzyme expression and of metabolite levels is not sufficient to indicate which metabolic pathway is utilized in any given cell and at any given time. Metabolome analysis based on stable labeled precursors and a systems modeling approach are the tools that are being developed, to describe how cells regulate their global metabolism.^8, 28, 29, 30^ Many maps of cancer metabolic rewiring (CMR) are reported in the literature,^31, 32, 33^ but they do not dedicate specific attention to the redox balance. The map we present in Figures 1 and 2 does so.

If we consider only the carbon flux, then Glutamine (Gln) is taken up by the cell through a transporter (Slc1a5), whose expression is stimulated by several oncogenic signaling pathways.^11, 34^ Then Gln is deaminated to glutamate (Glu) by cytoplasmic GLS1 and transferred by Slc25a11 mitochondrial carrier into the mitochondrial matrix. Otherwise, Gln may be transported into mitochondria, where it may be deaminated to Glu by GLS2. It is interesting to recall that Myc controls GLS1 expression,^14^ while p53^35, 36^ and p53-family members control GLS2.^37, 38, 39^ In turn, glutamate dehydrogenase (GDH) converts glutamate into α-ketoglutarate (Akg). This reaction of reductive carboxylation is performed in mitochondria by isocitrate dehydrogense enzyme (IDH) 2, but may be performed also in the cytosol by IDH1 Two pathways have been described for the Akg utilization in cancer cells, one counterclockwise (pathway B) and the other clockwise (pathway A). In pathway B (Figure 1), Akg is carboxylated to Isocitrate (IsoCit), which is converted into Citrate (Cit) and then exported into the cytoplasm. The cytosolic enzyme ATP-citrate-lyase (ACL) splits it to oxalacetate (Oaa) and Acetyl-CoA (AcCoA). AcCoA is then utilized as a building block for lipid biosynthesis.^40, 41^ In pathway A, Akg follows normal TCA cycle steps until Oaa, which is then converted into aspartate (Asp) by aspartate transaminase (GOT2) and exported into the cytoplasm. In turn, Asp may be transformed into Asparagine (Asn) and Arginine (Arg) to be used for protein synthesis.

## Involvement of NADH and NADPH in CMR

Many reactions of pathways A and B (Figure 1) are red/ox reactions, which are strongly affected by the availability of the two specific coenzymes NAD(H) and NADP(H). With regard to the reactions that take place in the mitochondrial matrix, as previously recalled, Complex I dysfunction is a fairly common event in cancer cells,^42, 43^ but other mutations can occur in the TCA cycle enzymes, fumarate hydratase (FH) and succinate dehydrogenase (SDH),^44, 45, 46^ or in other complexes of the electron transfer chain (ETC), such as Complex III.^47^ All these alterations have been shown to promote glutamine utilization by reductive carboxylation.^41, 47, 48^ An *in vitro* analysis of this reaction indicates that, for adequate citrate production to occur, through isocitrate dehydrogenase (IDH)-catalyzed reductive carboxylation, a high mitochondrial NADPH/NADP^+^ ratio is required.^49^ From this standpoint, although pathway B appears to be redox balanced, involving two reactions, the first one (GDH) generating NADPH and the other one IDH which consumes it, it is not surprising that the enhanced utilization of glutamine requires, *in vivo,* the activity of nicotinamide nucleotide transhydrogenase (NNT), a mitochondrial enzyme that transfers electrons from NADH to NADPH.^50^ In addition, in cancer cells with defective FH or with Complex III dysfunction, the oxidation of Akg in the TCA cycle (path A of Figure 1) is required to sustain concomitantly reductive carboxylation to Isocitrate, thereby indicating an overflow of reducing equivalents to be used for conversion of Akg into Isocitrate.^48^ While succinate accumulates in FH defective cancer cells, it does not do so in cancer cells harboring a defective Complex I.^48^ In the latter cells, each molecule of Akg is converted into Oaa producing two molecules of NADH and one molecule of FADH_2_. As previously indicated, NADH may be converted into NADPH by NNT, powering the reductive carboxylation reaction, or it may feed the dysfunctional ETC to produce ATP and consume oxygen. The residual activity of the ETC in K-ras transformed fibroblasts is around 50% of normal cells due to decreased Complex I activity and different expression levels of several OXPHOS genes.^10^ Consistent with these data, Hu *et al*^9^ have shown that *in vitro* activation of K-ras^G12V^ caused a disruption of Complex I. Specifically, using the SILAC (stable isotope labeling with amino acids in cell culture)-based mass spectrometry method, the authors have observed decreased levels of several Complex I components (NDUFA2, NDUFA4, NDUFA5, NDUFA11, NDUFA12, NDUFA13, NDUFB4, NDUFB6, and NDUFB7).^9^ To date, several interesting works have shown the link between oncogenic K-ras and mitochondrial dysfunction of cancer cells.^51, 52, 53, 54^ Moreover, this connection probably causes the metabolic reprogramming toward glutamine utilization in the TCA cycle to maintain cellular redox homeostasis.^8, 54, 55^ In conclusion, the inactivation of components of the ETC or the TCA cycle (characteristic of most cancer cells) sets the conditions which are able to promote glutamine utilization by reductive carboxylation. In fact, it leads to an increase in NADH/NAD^+^ in the mitochondrial matrix, which, by action of NNT, determines an increase in NADPH/NADP^+^, required to sustain reductive carboxylation of Akg.

Although many findings are available on the biological responses elicited by glutamine utilization in cancer cells, the fate of the products of glutamine metabolism, citrate and aspartate, that are exported from the mitochondria to the cytoplasm, is in part undetermined as yet. We present below a new interpretation that supports the notion that it is glycolysis, which sustains the reducing equivalents for a complete glutamine utilization.

In cancer cells, the expression of ACL is strongly upregulated^56^ and reductive glutamine metabolism, catalyzed by IDH1 and IDH2, results in an enhanced lipid biosynthesis.^41^ Related to this, overexpression of fatty acid synthase confers growth and survival advantages on cancer cells,^57^ while, on the contrary, inhibition of ACL suppresses tumor cells growth.^58^ Taken together, these findings indicate that a sustained production of lipids from AcCoA derived from glutamine takes place in cancer cells and is necessary to support their growth and survival.

Which are the metabolic sources of the NADPH required to convert AcCoA into lipids? In actively proliferating cells, the oxidative pentose phosphate pathway (PPP) has long been considered to be the major source of NADPH, with malic enzyme also being important in some cell types.^11, 59^ Recently, it has been reported that a comparable contribution comes from the Tetrahydrofolate (THF)-dependent pathway.^60^ This pathway stems from serine, whose production from the glycolytic intermediate 3-Phosphoglyceric acid (3Pga) requires phosphoglycerate dehydrogenase (PHGDH), an enzyme that generates NADH and whose function is strongly stimulated in many cancer cells (Figure 2).^61, 62, 63, 64^ In the conversion from serine into glycine (stimulated in cancer cells) methylenetetrahydrofolate is formed, whose oxidation generates NADPH (Figure 1).^64^ Quantitative flux analysis indicates that the synthesis of lipids is the pathway that utilizes a large share of NADPH production.^60^ By tracing hydrogen in compartmentalized pathways that utilize NADPH as a cofactor, fluxes of reactions that take place either within mitochondria or cytosol have been estimated.^65^ This interesting new metabolomic method, allowing cytosolic and mitochondrial NADPH compartment identification, has shown that the majority of mitochondrial NADPH and glycine is generated by mitochondrial serine hydroxymethyltransferase (SHMT2) and methylenetetrahydrofolate dehydrogenase 2 (MTHFD2). Consistent with Lewis and colleagues, a mathematical model of folate metabolism compartmentalization has identified in embryonic tissues and cancer cells, that SHMT2 and MTHFD2 mainly supported cytosolic purine and pyrimidine synthesis *via* the export of formate.^66^ Moreover, a very recent work has shown that SHMT2 was activated in cancer cells with oncogenic myc under hypoxic condition and knockdown of SHMT2 showed a reduced cellular NADPH/NADP^+^ ratio.^67^ Following this approach, it will be possible to investigate whether NADH generated by PHGDH is converted into NADPH by the combined activity of malate dehydrogenase (MDH1) and malic enzyme (ME1) (Figure 2). If this is the case, then one may answer the question whether the reducing power generated by the serine/glycine glycolitic diversion is exploited to increase the NADPH/NADP^+^ ratio, thereby stimulating non-essential amino-acid (NEAA) synthesis and increasing the reduced/oxidized glutathione ratio (GSH/GSSG) required for ROS quenching (Figure 2).

It is well known that the large majority of cancer cells are unable to grow only on glutamine, but require active glucose metabolism *via* glycolysis^23^ and PPP.^68^ The described serine/glycine diversion of glycolysis may then be viewed as a means for promoting glutamine utilization that fuels growth and survival of cancer cells.

## Role of CMR for Growth and Survival

**Figure 3:**
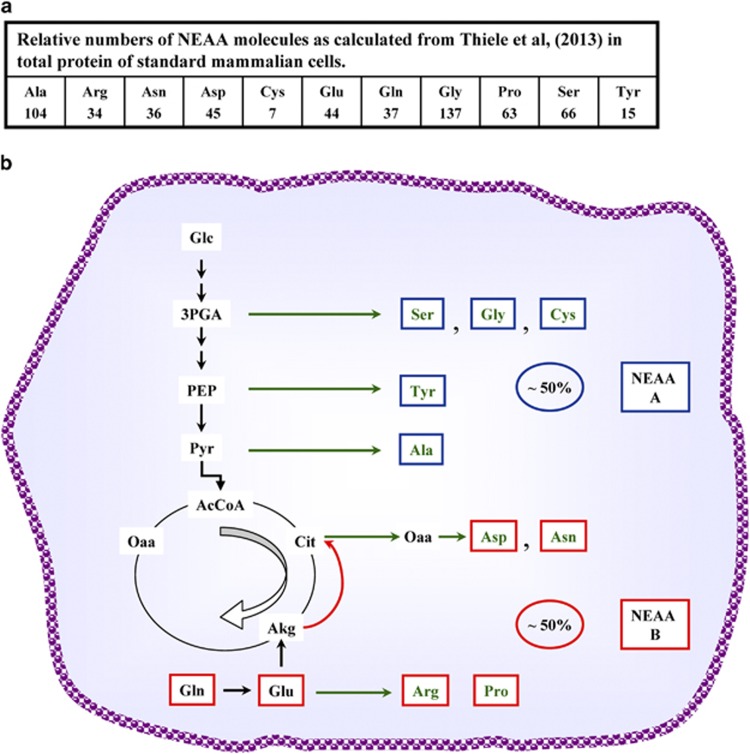
Schematic representation of NEAA synthesis. (**a**) Schematic fractional contribution of each amino acid in total protein and the corresponding number of molecules calculated for each NEAA that have to be obtained from metabolism. (**b**) Schematic representation of NEAA synthesis derived from glutamine utilization in cancer cells

To better understand the physiological role of CMR, it is interesting to define the supply of building blocks for growth that comes from each of its two processes: glycolysis and glutamine utilization by reductive carboxylation. Given that proteins are the largest cellular macromolecular components, about 70% of a dry weight of 500 pgr per cell, for standard mammalian cells,^26^ we utilize the reported fractional contribution of each amino acid in total protein and calculate the corresponding number of molecules for each NEAA that has to be obtained from metabolism (Figure 3a). The assignment to known synthetic pathways shows that at least 50% of the amino acids required for ‘*de novo*' protein synthetic activity of cancer cells derives from glutamine metabolism (Figure 3b). Due to the lack of specific tracing experiments, the amino acids derived from pyruvate have been assigned to the glycolytic pathway, although pyruvate may also be produced from glutamine (Figure 1). Of course this is only a crude approximation of the role of glutamine utilization in cancer cell growth, since glutamine provides also a large aliquot of nucleotides and lipids.

Proteome-wide analysis of protein turnover rates in growing mammalian cells has shown that most proteins have half-lives ranging from minutes to a few hours. Hence, a large part of protein biosynthetic activity is devoted to maintaining steady-state levels and not to producing proteins for the new cells.^69^ In this context, the process of mitophagy (autophagic elimination of mitochondria), promoted by excess nutrient availability, and resulting in mitochondrial dysfunction and depolarization,^70^ is of particular interest. Fragmentation of mitochondria, which precedes mitophagy, is observed in fact in cancer cells and takes place when glucose and glutamine uptake are stimulated.^59^ As for ATP utilization, the amount spent to sustain ‘*de novo*' cell growth is much less than that required for basal cellular maintenance.^61, 62^ The amino acids derived from extensive protein degradation may be recycled or enter the urea cycle producing polyamines and proline, both found to be upregulated in cancer cells.^71, 72^

Considering that the amino acids derived from glutamine are the ones obtained from Oaa and Akg, which are also intermediates of the TCA cycle, what is the physiological advantage for cancer cells to synthesize amino acids using glutamine by reductive carboxylation as compared with normal cells that derive them by using skeleton carbon from glucose and amino groups from glutamine?

The first obvious difference is to have immediately available Gln and Glu from the environment, thereby sparing cells their biosynthesis starting from Akg, produced by the TCA cycle. The second one is that practically all Gln, no matter which metabolic route is followed, is converted to Oaa. Accordingly, NEAA (B) shown in Figure 3b may be produced at a fast rate.

Moreover, the CMR ensures that the rate of glutamine utilization is coordinated with the stimulated rate of glycolysis, which characterizes cancer cells. Glycolysis in fact has a master control over the glutamine pathway, exerted by the regulation of the serine/THF diversion, with the rate of NADPH production (and possibly NADH) being exploited in several steps of the glutamine pathway. Normal cells instead control the rate of production of NEAA in a more rigid way: it is the rate of refilling of Oaa from pyruvate that allows the utilization of the intermediates Akg and Oaa away from the TCA cycle.

Taken together, the findings discussed so far point, for the first time, to a strong regulatory role of NAD(H) and NADP(H) metabolism in sustaining cancer cell growth.

## NADH and Tumor Aggressiveness

**Figure 4:**
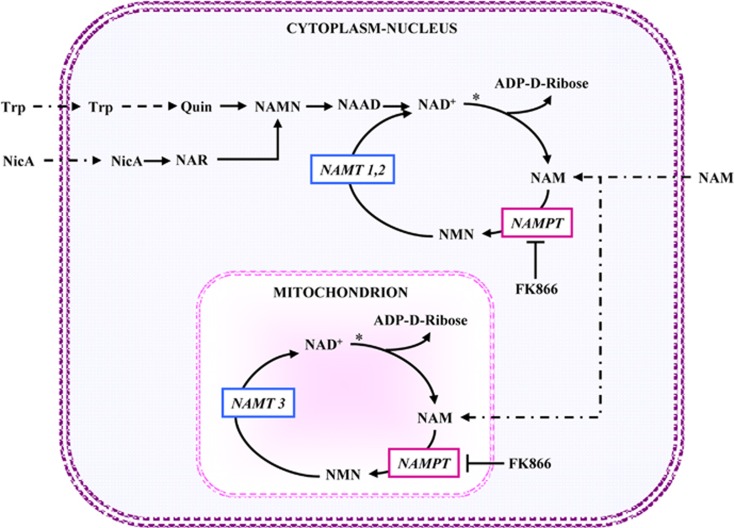
Map of the NAD±biosynthetic pathway. To clarify the interpretation of the ‘*in vivo*' experiments described in the test, the NAD^+^ biosynthetic pathway is schematically reported: NAD^+^ can be synthesized from tryptophan (*de novo* synthesis) or nicotinic acid -NicA- or nicotinamide -Nam- (salvage pathway). NAD^+^ synthesis *via* tryptophan is performed by several reactions that lead to quinolinic acid (Quin) synthesis: this is next converted into nicotinic acid mononucleotide (NAMN) and NAMN to desamido-NAD (NAAD) and eventually into NAD^+^. Diversely, in the major salvage pathway of NAD^+^ synthesis, NAM can be converted into nicotinamide mononucleotide (NMN) by nicotinamide phosphoribosyltransferase (NAMPT) and NMN is further converted into NAD^+^ by NMN adenylyltransferase (NMNAT 1 and 2 nuclear and cytoplasmic isoforms respectively, NMNAT3 mitochodrial isoform). The asterisk indicates a reaction, catalyzed by NAD^+^ glycohydrolase, which bears close similarity with the family of ADP ribosyl cyclases. However, all the NAD^+^-transforming enzyme families mentioned above (i.e., Mono ADP-ribosyl transferases, PARP_s_, sirtuins) share with ADP ribosyl cyclases/NAD^+^ glycohydrolases the property of releasing NAM from NAD^+^

The relevance of NAD metabolism in tumor development is supported by the results of a number of experiments, in which the availability of NAD(H) has been manipulated by genetic or biochemical means. Recently, considerable attention has been focused on NAMPT (Figure 4) as a potential therapeutic target, since several reports have shown its overexpression in several kinds of^73, 74^ and, conversely, its downregulation by genetic or chemical means suppresses tumor cell growth *in vitro* and in tumor xenografts as well.^75, 76, 77^ One of the most widely used inhibitors of NAMPT is FK866, which is under development as an anticancer drug.^24, 78^

When human breast cancer cells, defective in Complex I activity, are transfected to express yeast NADH dehydrogenase, Ndi1, their ability to oxidize mitochondrial NADH is restored and, at the same time, their tumor forming ability in xenografts is substantially reduced.^79^ Given that the Ndi1 expression enhances the NAD^+^/NADH ratio in whole cells and mitochondria, the effect was analyzed by supplying NAD^+^ precursors, NicA and NAM^80^ to the same Complex I-defective breast cancer cells. The tumor forming ability of xenografts is dramatically reduced by treatment with NicA and NAM, both able to refill the level of NAD^+^ in mitochondrial and in cytoplasmic/nuclear compartments (Figure 4). Moreover, the supplying of NAD^+^ precursors in drinking water, after primary breast cancer removal in animal models, increases survival.^79^ Consistent with the previous observations, these findings confirm that dysfunction of the ETC or of TCA cycle enzymes is required to trigger glutamine utilization by reductive carboxylation and suggest the possibility of a regulatory role of NADH/NAD^+^ ratio in the onset of glutamine utilization.

Treatment with FK866 strongly reduces the NAD^+^ available to the glyceraldehyde 3-phosphate dehydrogenase (GAPDH). This inhibition, on the one hand, leads to an accumulation of glycolytic intermediates upstream of the inhibited GAPDH step, with an increased flux into the PPP^68^ and, on the other hand, reduces the carbon flow into the serine/THF pathway and, in a less stringent way and in the presence of non-limiting glutamine, in the TCA cycle as well.^77^

The dependence of cancer cell growth on NAD(H) seems to be far more complex than previously described. For instance, it has been shown that the decrease in NAD^+^ levels, obtained by inhibiting with shRNA the expression of NAMPT, could make tumor cells more aggressive,^79^ while the treatment with FK866, that targets the same enzyme,^81^ leads to cancer cell death both *in vitro* and *in vivo*.^76^ This discrepancy supports the notion that FK866, besides featuring an inhibitory effect on NAMPT, could have additional cytotoxic properties on cancer cells.^79^

Moreover, recent data have demonstrated that pharmacological inhibition of NAMPT by FK866 can be counterbalanced by rescue of NAD^+^ production from precursors other than NAM, notably NMN and Nicotinamide riboside (NR).^82^ Specifically, different FK866-treated tumor cells are affected by two ectoenzymes that are able to produce NAD^+^ precursors. CD73 (generating NR from NAD^+^ and NMN) enables, while CD38 (generating NAM that cannot be converted into NAD^+^ because of NAMPT inhibition) impairs intracellular NAD^+^ biosynthesis and consequently cell viability.

Finally, to evaluate more critically the findings observed after FK866 treatment, it should be clarified whether the mitochondrial pool of NAD(H) is sensitive to FK866, given that literature data do not agree on this point.^80, 83^

## Conclusions and Future Perspectives

A lot of attention has been devoted, in recent years, to signaling and transcriptional events that promote cancer insurgence and progression, in an effort to develop a large number of new, target-oriented anticancer drugs. Unfortunately, the efficacy of these drugs in patients is often temporary, due to the frequent insurgence of molecular mechanisms of drug resistance.^84^ The recent awareness that many signaling pathways converge to a common bottom line, the metabolic rewiring, characterized by mitochondrial dysfunction, enhanced glycolysis and stimulated glutamine utilization through a reductive carboxylation reaction, offers the possibility of developing a new strategy for anticancer drug discovery.^85^ In fact, although a study of Marin-Valencia *et al* has shown that tumors, derived from transplantation of glioblastoma cells, used glucose to sustain anabolic processes accumulating glutamine,^86^ several works have demonstrated the efficacy of glutaminase inhibitors as BPTES (bis-2-(5-phenylacetamido-1,2,4-diathiazol-2-yl) ethyl sulfide) and CB-839 both *in vitro* and *in vivo*.^21, 87, 88, 89, 90, 91^ Moreover, since the metabolic differences are mainly due to different kinds of tumor phenotype, a detailed metabolic profiling of human tumors should be performed to clarify the cancer types in which CMR takes place. For these cancer types, the concept map of the cancer rewired metabolism presented in this paper offers a rational basis for the development of a drug discovery strategy that aims to target rate-limiting nodes of the CMR. Moreover, this drug strategy to become really effective requires the evaluation of a number of quantitative aspects.

First of all, the utilization of various metabolomics techniques, such as gas chromatography-mass spectrometry (GC-MS) or liquid chromatography (LC-MS) combined with stable isotope tracers, in order to perform metabolic flux reconstruction and quantitative evaluation of enzyme function by metabolic flux analysis (MFA).^8, 29, 41, 92, 93, 94^ In addition, this experimental/computational approach should permit the estimation of the fluxes in the various pathways (especially those less well known at the moment, such as the utilization of the reducing power produced by the serine/THF pathway^60, 65^ in various cancer cells *in vitro* and possibly also *in vivo*).^95^ Given that present day technologies may accurately describe the metabolic profiles even in formalin-fixed paraffin-embedded tumor biopsy samples,^96^ retrospective studies are feasible. More information on the role of mitochondrial and cytoplasmic NAD(P)H/NAD(P) ratio in sustaining cancer metabolic rewiring may also be obtained by treatment with NAD pathway precursors or inhibitors, taking into careful consideration the aspects presently under debate. Development of quantitative dynamic models^97^ of the rewired cancer metabolism and of its regulation would add predictive ability to the new understanding (Box 1).^85^

Since the metabolic conditions required for tumorigenesis are Complex I dysfunction and enhanced glucose and glutamine utilization, the concept map presented herein may also offer a new explanation for the reported increase of cancer incidence with aging.^98^ The reduction of Complex I activity characterizes the decline of metabolism during aging;^99^ this event originates a favorable ground for the oncogenic expression of somatic mutations able to stimulate nutrient uptake. Without the mitochondrial dysfunction related to aging (or to other causes), the simple stimulation of glucose and glutamine uptake might turn out to be non-tumorigenic. A deeper understanding of cancer metabolic rewiring proposed in Figures 1 and 2 and of its regulation may clarify whether sirtuins have a prevalent role in age-induced tumorigenesis, as proposed by Wu and colleagues,^100^ and may indicate ways to reactivate Complex I activity to prevent both aging and age-induced tumorigenesis.

## Box 1. Computational models and design principles of cancer metabolic rewiring.

A deeper understanding of cancer metabolic rewiring requires the development of computational models^85, 97^ that should consider at least all the metabolic reactions encompassed by the concept map of Figures 1 and 2.

A number of studies, generally dealing with specific pathways, such as glycolysis or TCA cycle, are reported in the literature. Two approaches are prevalent: quantitative mathematical models or genome-wide metabolic models, both of which present limitations as well as new insights. Of interest are, for instance, a genome-wide study of the Warburg effect that links metabolic steps to cancer cell migration^101^ and a mathematical modeling of the serine/glycine conversion rate in the NCI60 tumor-derived cell lines that shows the contribution of glycine in purines, ATP, and NADPH production in cancer cells.^102^ The development of reliable, quantitative models linking metabolic rewiring to enhanced growth and survival of cancer cells is a next step. In the meantime, it may be of interest to comment on design principles that differentiate cancer cells from normal ones. It is often said that cancer cells have an inefficient metabolism since they only partially use the chemical potential of glucose, carrying on lactic fermentation and excreting lactate,^103^ while normal cells are more efficient since they fully utilize glucose in glycolysis and TCA cycle. But the situation is reversed for glutamine (the other relevant substrate for mammalian cells both ‘*in vitro*' and ‘*in vivo*'). Cancer cells use both carbon and nitrogen of glutamine, while normal cells use only nitrogen and excrete glutamic acid.^8^

In cancer cells, the production of reduced coenzymes for each glucose molecule entering either in the PPP or in serine/glycine pathway is different: for two molecules of NADPH formed in the first pathway, there are two of NADH and two of NADPH in the second one.

With regard to the relation of metabolism with growth, if one recalls that proteins are the largest component of biomass, it has to focus on the metabolic pathways bringing to amino acids (Figure 3). In normal cells, the rate of utilization of amino acids from TCA cycle is limited by the rate of refilling of Oaa to the TCA cycle from pyruvate and by availability of conditions that are going to affect the efficiency of TCA cycle as provider of amino acids (favorable ADP/ATP ratio, functionality and rate of execution of the electron transfer chain and availability of oxygen). In cancer cells, once the few enzymes of pathways A and B of Figures 1 and 2 are available, the rate of production of NEAA B depends only upon the ability of PPP and/or of serine/glycine pathway to regenerate NADPH, while the rate of production of NEAA A is expected to follow the enhanced rate of glycolysis.

In conclusion, in cancer cells glycolysis with its related pathways appears to have a master feed-forward control over glutamine utilization pathway, a condition that may be relevant in sustaining tumor forming ability ‘*in vivo*'.

## Abbreviations

1,3BPGA: bisphosphoglycerate
3PGA: 3phosphoglycerate
6PGL: 6-phosphogluconolactone
AcCoA: acetyl-CoA
ACL: ATP citrate lyase
Akg: *α*-ketoglutarate
Ala: alanine
Arg: arginine
Asn: asparagines
Asp: aspartate
CH^+^-Thf: 5-methylenetetrahydrofolate
CH_2_-Thf: 5,10-methylenetetrahydrofolate
Cit: citrate
F1,6BP: fructose 1,6 biphosphate
FFA: fatty acids
Fum: fumarate
G6P: glucose 6-phosphate
GA3P: glyceraldehyde 3-phosphate
GDH: glutamate dehydrogenase
Glc: glucose
Gln: glutamine
Glu: glutamate
Gly: glycine
GOT: glutamic-oxaloacetic transaminase
GSH: glutathione reduced
GSSG: glutathione oxidized
Isocit: isocitrate
Lac: lactate
LDHA: lactate dehydrogenase
Mal: malate
MDH 1 and 2: malate dehydrogenase
ME: malic enzyme
MTHFD1 and 2: methylenetetrahydrofolate dehydrogenase (NADP^+^ dependent)
NEAA: non-essential amino acids
NNT: nicotinamide nucleotide transhydrogenase
Oaa: oxalacetate
PDH: pyruvate dehydrogenase
PDHKs: pyruvate dehydrogenase kinases
PPP: pentose phosphate pathway
P-Pyr: 3-phosphohydroxypyruvate
Pyr: pyruvate
R5P: ribose 5-phosphate
ROS: reactive oxygen species
Ser: serine
SHMT1 and 2: serine hydroxymethyltransferase
Succ: succinate
